# Exploring the significance of fatty acid-binding protein 4 levels as a biomarker for autism spectrum disorder: a cross-sectional study

**DOI:** 10.1186/s12888-026-08295-4

**Published:** 2026-06-22

**Authors:** Asmaa Wafeeq Abdelaziz, Mohammad Mostafa Alkherkhisy, Heba-Allah Ramadan El-Sheikh, Salwa Amin Abd Elhamid

**Affiliations:** 1 Pediatrics Department, Faculty of Medicine, Ain Shams University, Abbassya Square, Cairo, Egypt; 2 Medical Microbiology and Immunology, Faculty of Medicine, Al-Azhar University, Cairo, Egypt

**Keywords:** Autism spectrum disorder, FABP4, Biomarkers, Intellectual disability

## Abstract

**Background:**

Lipid metabolism and its regulatory molecules, especially adipokines, have gained significant attention in recent pathogenesis research of autism spectrum disorder (ASD).

**Aims:**

To evaluate serum fatty acid-binding protein 4 (FABP4) levels as a potential biomarker for autism spectrum disorder (ASD), comparing them to levels in children with intellectual disabilities and typically developing peers, and to determine whether variations in FABP4 can effectively differentiate children with ASD from those with intellectual disabilities.

**Methods:**

A comparative cross-sectional study was conducted with 90 children aged 3 to 12 years, divided into three groups: 30 children with autism spectrum disorder (ASD), 30 with Intellectual Disability (ID), and 30 healthy controls. Serum samples were collected and analyzed for FABP4 levels. Demographic characteristics, cognitive function and severity of ASD disease were collected and analyzed in relation to the FABP4 level.

**Results:**

Mean serum FABP4 levels were significantly lower in both ASD and ID groups compared with controls (*p* < 0.001). FABP4 demonstrated excellent diagnostic performance for differentiating ASD from controls (AUC = 1.00, cut-off < 3.99 ng/mL, 100% sensitivity and specificity) and ID from controls (AUC = 0.989, cut-off < 3.77 ng/mL, 96.7% sensitivity and 100% specificity). However, FABP4 showed limited ability to distinguish ASD from ID (AUC = 0.63, *p* = 0.090). No significant correlation was found between FABP4 levels and age, BMI, and ASD severity (*p* > 0.05).

**Conclusion:**

The data suggest that FABP4 may serve as a marker of neurodevelopmental dysfunction, rather than functioning as a specific biomarker for diagnosing autism.

**Clinical trial number:**

Not applicable.

## Introduction

Neurodevelopmental disorders (NDDs) represent a diverse group of conditions that profoundly influence brain development and function, exhibiting a remarkable range of genetic and clinical variability. One of the most complex and challenging among these is autism spectrum disorder (ASD). Although the exact causes of ASD are still unclear, increasing evidence suggests a complex interplay between genetic, environmental, and metabolic factors in its development. Among these factors, lipid metabolism and its regulatory molecules, especially adipokines, have gained significant attention in recent research.

Adipokines, powerful bioactive proteins predominantly secreted by white adipose tissue (WAT), exert a profound influence that exceeds ordinary peripheral metabolic regulation. Remarkably, they are also present in the central nervous system (CNS), where they interact with specific receptors. Their role is predominant in regulating neuroinflammation and oxidative stress; two critical physiological processes complexly linked to neurodegeneration and various neurodevelopmental disorders [1].

Notably, fatty acid-binding proteins (FABPs) are emerging as important players in this area. Previous studies have shown that they play significant roles in the transportation and metabolism of fatty acids [2, 3]. Adipocyte fatty acid-binding protein, commonly referred to as FABP4 or adipocyte protein 2 (aP2), is a vital member of the fatty acid-binding protein family [4]. Emerging research has identified FABP4 as an adipokine that significantly impacts metabolic disorders including obesity, type 2 diabetes, and atherosclerosis. It intricately modulates lipid-mediated signalling pathways that govern the secretion of pro-inflammatory cytokines, establishing a direct link between lipid metabolism and inflammation [5].

While FABP4 is primarily associated with adipose tissue and macrophages, its presence in the central nervous system (CNS) cannot be overlooked. Within the CNS, FABP4 is found in neurons and glial cells, where it likely plays a critical role in brain lipid metabolism and the regulation of neuroinflammation. By influencing these vital pathways, FABP4 may significantly contribute to the onset and progression of neurological disorders, including Alzheimer’s disease and autism spectrum disorder (ASD) [6].

While numerous studies have explored FABP4 levels in metabolic disorders and demonstrated its significant role in their etiopathogenesis, there are no studies that have examined its possible role in the pathogenesis of pediatric neurodevelopmental disorders, except for a recent study in Japan suggesting that FABP4 may play a innovative role in the pathogenesis of ASD [7]. Thus, our study aims to by examine FABP4 levels as a biomarker of adipobrain in children with ASD, in comparison to peers with intellectual disabilities and typically developing children. Additionally, we aim to determine whether variations in FABP4 levels can effectively distinguish children with ASD from those with intellectual disabilities.

## Participants and methods

### Study design and participants

This comparative cross-sectional study was conducted after receiving approval from the local ethical committee and parental consent. The Data were collected by the investigators, analyzed by statisticians and interpreted by the authors. This study included 90 participants divided into three groups: 30 children with autism spectrum disorder (ASD), 30 children with Intellectual Disability (ID), and 30 age-, sex-, and BMI-matched healthy controls. The ASD group included 21 males and 9 females, while the ID group comprised 19 males and 11 females. The control group included 13 males and 17 females. Eligible children with ASD and ID were clinically diagnosed based on the Diagnostic and Statistical Manual of Mental Disorders, Fifth Edition (DSM-5) [8]. Exclusion criteria included children with other neurodevelopmental, psychiatric, genetic, neurological, or chronic disorders.

A sample size of at least 30 children diagnosed with autism spectrum disorders, 30 children diagnosed with intellectual disability and 30 healthy controls was deemed necessary for detecting 100% power, at alpha error 0.05, by using Power analysis and sample size software (PASS 15) (Version 15.0.10) for sample size calculation.

### Procedure and measures

Data on the demographic characteristics of participants, such as age, sex, and BMI, were collected along with relevant medical histories, including perinatal and family histories of similar conditions. Possible confounding variables, like age and BMI, were identified from previous literature as factors that could influence the level of FABP4. To address these confounding factors, comparable groups were used based on age and BMI.

#### Childhood Autism Rating Scale (CARS)

The Childhood Autism Rating Scale (CARS) is a behavioral rating scale consisting of fifteen subscales that was developed to distinguish children with autism from those with other developmental abnormalities. The scale has fifteen items, each of which addresses a distinct trait frequently linked to autism. Patients with CARS scores of 30–36 were categorized as mild-to-moderately autistic, whereas those with scores of 37–60 were categorized as severely autistic [9].

### Laboratory procedures

#### Sample preparation

After an 8-hour fast, venous blood was drawn from patients and controls between 8:00 and 10:00 in the morning. Serum vacuum tubes with clot activator were used for direct sampling, and the tubes were left at room temperature for 20 to 30 min before centrifugation to allow blood coagulation. The samples were then centrifuged at 3,000 × g for 15 min at 8 °C. The serum was separated and separated into aliquots, and all the serums were kept for further use at -80 °C.

#### ELISA assay for the measurement of serum levels of FABP4

A commercial Enzyme-Linked Immunosorbent Assay(ELISA) kit Cat. No: E2036Hu (Bioassay Technology Laboratory, FABP4 ELISA Kit, Shanghai, China) was used to check the FABP4 level in the serum samples.

The manufacturer’s instructions were followed in the preparation of the samples, tools, reagents, and standards dilutions. The assay process comprised two wells, one labeled with biotin or streptavidin- Horseradish Peroxidase (HRP) for the anti-FABP4 antibody and the other with no material. The following were then included in the conventional wells: Each well was treated with 50 µl of streptavidin-HRP and 50 µl of a ready standard solution that contained the labeled anti FABP4 antibody. Additionally, the following sample wells were to be tested: a 50-µl streptavidin-HRP, a 10-µl FABP4 antibodies, and a 40-µl sample. After covering and sealing the prepared plate and shaking it to ensure thorough mixing, it was incubated for 60 min at 37 °C. After thorough cleaning, 50 µl of solutions were added to each well. After properly cleaning, 50 µl of solution A (chromogen solution) and 50 µl of solution B (chromogen solution) were added to each well. After that, the dish was gently shaken to keep the mixture dark and at 37 °C for ten minutes. Finally, the stop solution was added to each well to stop the reaction. The Ooptical density (OD) readings were then read, and concentrations were computed within ten minutes. FABP4 levels were measured twice in each sample, and statistical analysis was performed using the mean of the two results. The sensitivity of the assay was 0.053 ng/ml, and the intra- and inter-assay coefficients of variance were 8% and 10%, respectively.

### Statistical analysis

Data were analyzed using the Statistical Package for the Social Sciences (SPSS), version 26.0 (SPSS Inc., Chicago, Illinois, USA). The quantitative data was presented as mean ± standard deviation (SD) for normally distributed variables, while non-normally distributed variables were reported as median with interquartile range (IQR). Qualitative variables were expressed as counts and percentages. To assess the normality of the data, the Kolmogorov-Smirnov and Shapiro-Wilk tests were employed. For comparisons between two means, the independent-samples t-test was utilized. For non-parametric data, the Kruskal-Wallis’s test was applied for multiple group comparisons, and the Mann-Whitney U test was used for two-group comparisons. A one-way analysis of variance (ANOVA) was conducted when comparing more than two means, followed by Tukey’s post hoc test for multiple comparisons. The Chi-square test and Fisher’s exact test were used for comparisons involving qualitative data, with the latter applied when the expected count in any cell was less than five. To evaluate the degree of association between two sets of variables, Spearman’s rank correlation coefficient (Rs) was calculated. A positive correlation indicates that an increase in the independent variable leads to an increase in the dependent variable, while a negative correlation suggests the opposite. Receiver operating characteristic (ROC) curve analysis was performed to evaluate the overall predictive performance of the studied parameter and to determine the optimal cut-off value, including its corresponding sensitivity and specificity.

A p-value of less than 0.05 (*p* < 0.05) was considered statistically significant, while p-values less than 0.001 (*p* < 0.001) were considered highly statistically significant.

## Results

### Characteristics of participants

The demographic data of the participants are summarized in Table 1. A total of 90 participants were enrolled, consisting of 60 patients divided into two groups: 30 children with autism spectrum disorder (ASD) (21 males and 9 females) with a mean Childhood Autism Rating Scale (CARS) score of 36.22 ± 6.02 (66.7% mild to moderate versus 33.3% severe autistic scores) and 30 children with Intellectual Disability (ID) (19 males and 11 females) compared to 30 typically developed age- and sex-matched controls (13 males and 17 females). The mean ages of the groups were 6.98 (± 1.47) years for the ASD group, 7.35 (± 1.33) years for the ID group, and 6.72 (± 1.60) years for the control group. No statistically significant differences were observed among the study groups regarding age (*p* = 0.251), sex (*p* = 0.092), BMI (*p* = 0.528), gestational age at delivery (*p* = 0.117), or history of NICU admission (*p* = 0.322). While intelligence quotient (IQ) scores demonstrated a highly statistically significant difference between the groups (*p* = 0.001). The mean IQ score was 54.57 ± 11.01 in the ASD group, 59.43 ± 10.49 in the ID group, and 86.83 ± 5.71 in the control group. Additionally, family history also showed a highly significant difference (*p* = 0.001); (56.7%) of cases in the ASD group and (33.3%) of cases in ID group had positive family history of neurodevelopmental disorders in comparison to control group.

**Table 1:** Comparison between groups according to sociodemographic and clinical characteristics

	Autism Group (*n* = 30)	ID Group (*n* = 30)	Control Group (*n* = 30)	Test value	*P*-value
Age(years) Mean ± SD	6.98 ± 1.47	7.35 ± 1.33	6.72 ± 1.60	1.406	0.251
Range	4–9	4–10	4–9		
Female	9 (30.0%)	11 (36.7%)	17 (56.7%)	4.773	0.092
Male	21 (70.0%)	19 (63.3%)	13 (43.3%)		
Mean BMI(kg/m2)	16.94 ± 3.02	17.44 ± 2.69	16.73 ± 1.51	0.643	0.528
Negative family history of NDD	13 (43.3%)	20 (66.7%)	29 (96.7%)	20.012	**0.001**
Positive family history of NDD	17 (56.7%)	10 (33.3%)	1 (3.3%)		
Pre-term	2 (6.7%)	0 (0.0%)	4 (13.3%)	4.286	0.117
Full-term	28 (93.3%)	30 (100.0%)	26 (86.7%)		
NICU admission (No)	25 (83.3%)	25 (83.3%)	24 (80.0%)	10.250	0.419
NICU admission (Yes)	5 (16.7%)	5 (16.7%)	6 (20.0%)		
IQ Score Mean ± SD	54.57 ± 11.01	59.43 ± 10.49	86.83 ± 5.71	13.254	**0.001**
Range	30–80	39–70	77–96		

Data are presented as mean ± standard deviation (SD)

### FABP4 level

**Fig. 1:**
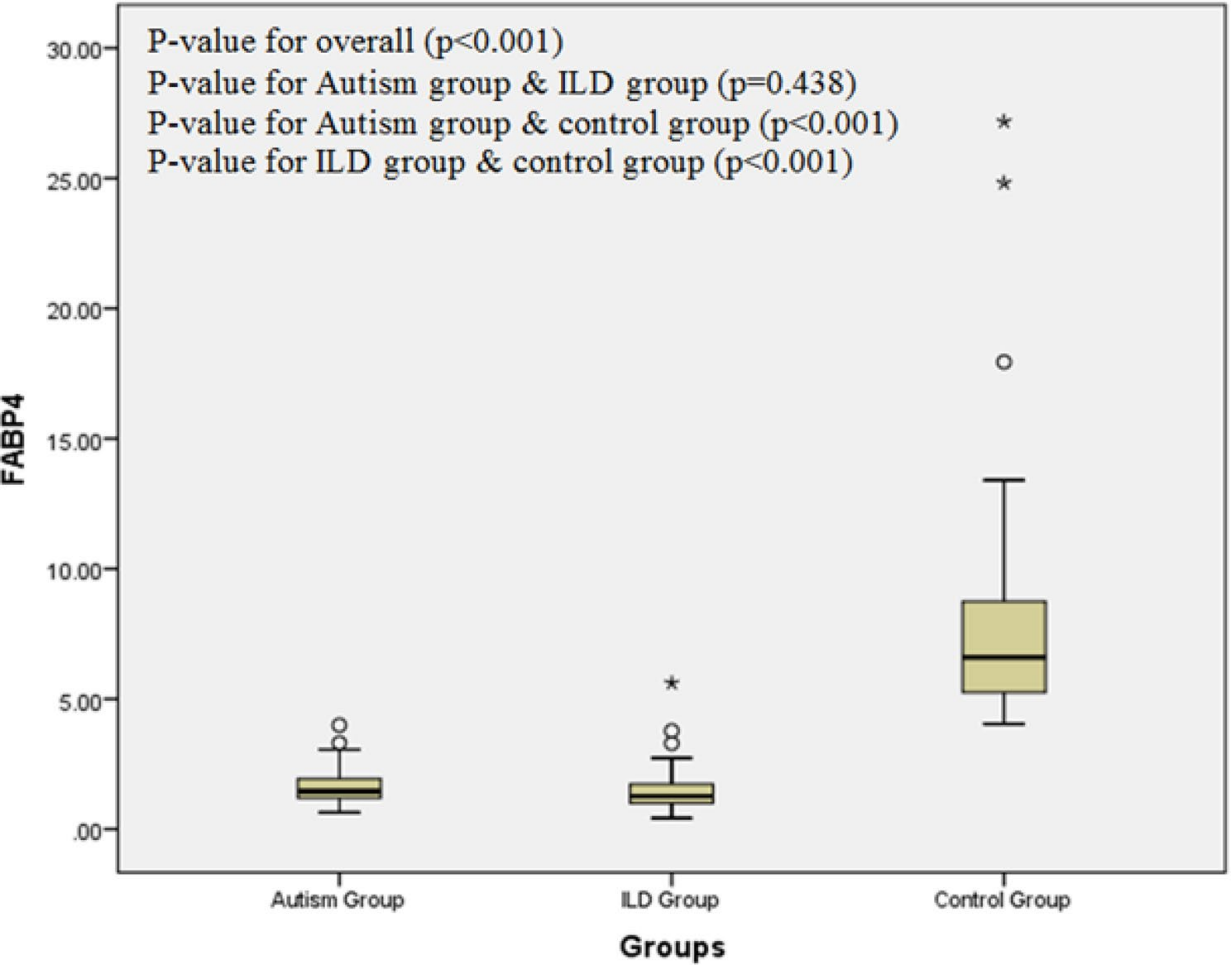
Box Plot of FABP4 level among studied groups

The ASD group showed statistically significantly lower median level of **FABP4** than the typically developed group (1.45 versus 6.60; *P* = 0.001, respectively). Similarly, the ID group showed statistically significantly lower median level of **FABP4** than the typically developed group (1.26. versus 6.60; *P* = 0.001, respectively), one control participant demonstrated an extreme FABP4 outlier value (27.18). Sensitivity analysis excluding this observation did not materially alter the overall results, and group differences remained highly statistically significant). However, there was no statistically significant difference in the marker level between ASD and ID groups (*p* = 0.438) (Fig. 1).

**Fig. 2:**
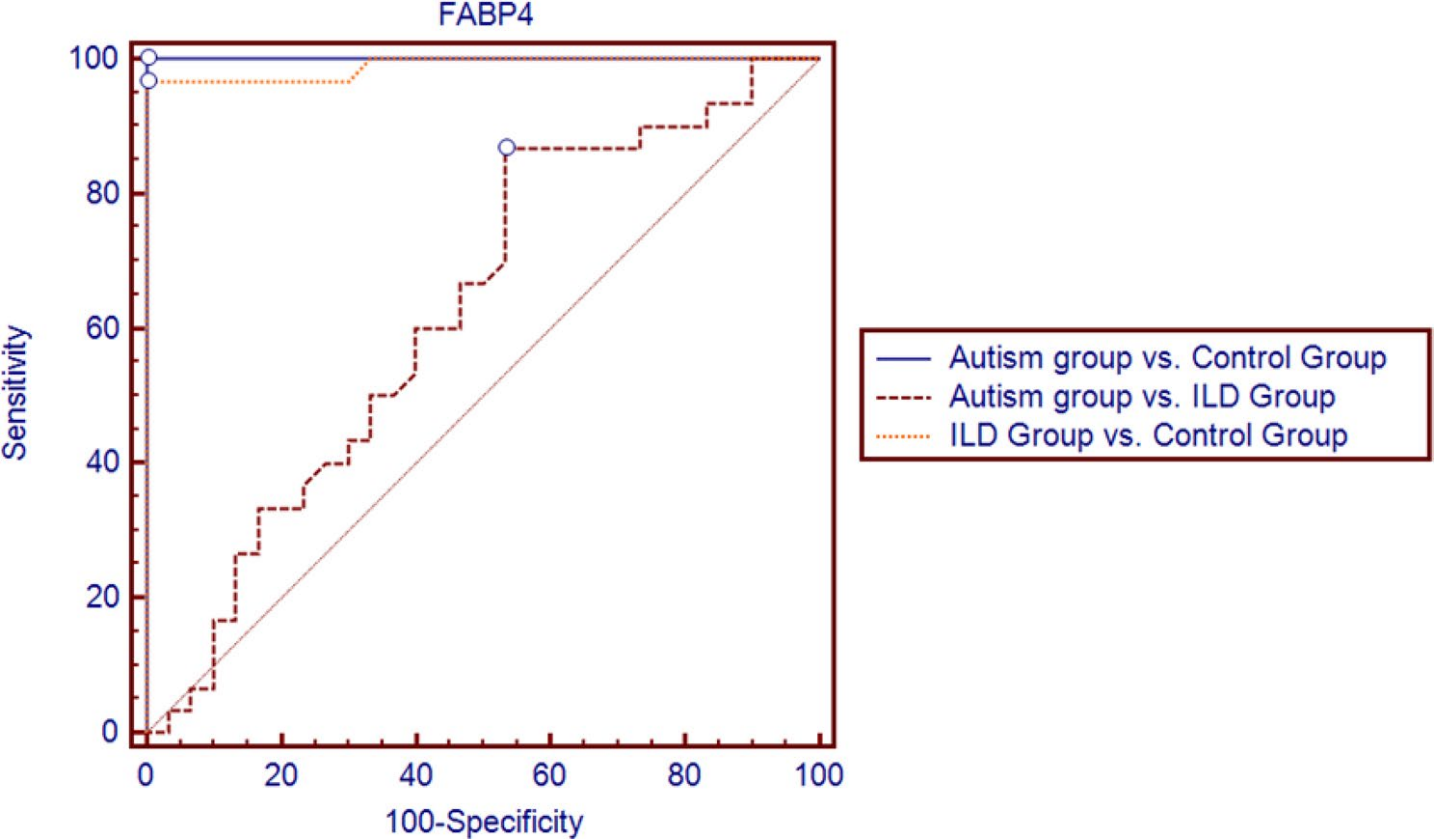
Receiver-operating characteristic (ROC) curve for diagnostic performance of FABP4 in discrimination of autism group and other group

ROC curve analysis demonstrated excellent diagnostic performance of FABP4 in differentiating ASD from controls, with an area under the curve (AUC) of 1.00 (95% CI: 0.94–1.00; *p* < 0.001). The optimal cut-off value was < 3.99 ng/mL, yielding 100% sensitivity and 100% specificity. For discrimination between the ID and control groups, the optimal cut-off value was < 3.77 ng/mL, with 96.7% sensitivity and 100% specificity, and an AUC of 0.989 (95% CI: 0.92–1.00; *p* < 0.001). However, FABP4 showed limited ability to distinguish ASD from ID, with a cut-off value of > 1.15, sensitivity of 86.7%, specificity of 46.7%, and an AUC of 0.63 (95% CI: 0.49–0.75; *p* = 0.090). (Fig. 2).

The levels of FABP4 did not differ between those who had positive or negative family history of neurodevelopmental disorders, similarly no difference was found in relation to gestational age at birth nor history of Nicu admission in all groups (*p* > 0.05) (Table 2).

**Table 2:** Relation between different variables and FABP4 in each group

Variables	FABP4 in ASD	Test value	*p*-value	FABP4 in ID	Test value	*p*-value	FABP4 in controls	Test value	*p*-value
Pre-term	1.23	0.997	0.318	N/A†	N/A†	N/A†	7.75	0.493	0.483
Full-term	1.51	0.997	0.318	1.26	N/A†	N/A†	6.33	0.493	0.483
Non-NICU admission	1.49	0.003	0.956	1.24	2.097	0.148	6.69	1.302	0.254
NICU admission	1.28	0.003	0.956	2.73	2.097	0.148	5.61	1.302	0.254
Positive family history	1.49	0.007	0.933	1.19	0.109	0.741	27.18	2.808	0.094
Negative family history	1.41	0.007	0.933	1.29	0.109	0.741	6.50	2.808	0.094

Using: U=Mann-Whitney test for Non-parametric data “Median (Interquartile range: IQR)”

Kruskal–Wallis was performed for Median (IQR) and Multiple comparison of Mann-Whitney test for Non-parametric data

Different capital letters indicate significant difference at (*p* < 0.05) among means in the same column

N/A†: No pre-term infants were reported in the ID group; therefore, statistical testing could not be performed

The positive family history subgroup in the control group consisted of a single participant (*n* = 1); therefore, the reported FABP4 value represents a single observation and should be interpreted cautiously

We did not observe any significant correlations between the levels of FABP4 and the age, BMI, and IQ score in all groups Additionally, no significant correlation between FABP4 levels and CARS in ASD group (*p* > 0.05) (Table 3).

**Table 3:** Correlation between FABP4 with different parameters

Variables	FABP4 in ASD		FABP4 in ID		FABP4 in controls	
	*r* value	*P*- value	*r* value	*P*- value	*r* value	*P*- value
Age(years)	-0.258	0.168	-0.009	0.961	0.030	0.877
BMI (kg/m²)	-0.287	0.124	0.067	0.724	0.026	0.890
IQ Score	-0.057	0.763	0.116	0.540	-0.060	0.753
CARS	0.265	0.156				

Using: Spearman’s rank correlation coefficient (rs)

BMI: Body Mass Index, FABP4: Fatty Acid Binding Protein-4

## Discussion

Autism Spectrum Disorder (ASD) is believed to arise from complex interactions between genetic and environmental factors, although the precise mechanisms involved in its development remain unclear [10, 11]. In this study, we examined the “adipose-brain axis” as a potential contributing factor to the pathophysiology of ASD. We specifically analyzed Fatty Acid Binding Protein 4 (FABP4) levels as a biomarker for fat metabolism in a sample of Egyptian children diagnosed with ASD. This group was compared to both age- and sex-matched children with intellectual disabilities (ID) and typically developing controls.

Children diagnosed with autism spectrum disorder (ASD) showed significantly reduced levels of FABP4 compared to those typically developing children, although there was no significant relationship identified with the severity of ASD. The diagnostic performance of FABP4 in this study (AUC = 1.00) clearly distinguishes between the ASD and control groups, with no overlapping values. We established a cutoff of less than 3.99, achieving 100% sensitivity and specificity. However, these results should be viewed cautiously because the distinct separation may stem from the specific characteristics of our cohort, including the homogeneous clinical traits of the ASD participants, strict selection criteria, and limited sample size, which might lead to non-overlapping distributions by chance. Additionally, the single-centre recruitment and possible population-specific factors could have influenced our findings. Validation in larger, independent populations is necessary.

These results are consistent with findings by [7] who noted that young children with autism (aged 2–6 years) had lower FABP4 levels than their typically developing peers, using a cut-off level of 12.7 ng/ml and achieving sensitivity and specificity values of 81.0% and 71.4%, respectively. Similar reductions in serum FABP4 levels have also been observed in patients with schizophrenia, which is often viewed as the adult manifestation of ASD. In research by [12], FABP4 levels were found to be decreased in the dorsolateral prefrontal cortex of postmortem brains from individuals with schizophrenia. Additionally, a study by [13] reported a substantial 40% decrease in FABP4 levels in scalp hair follicles from individuals with schizophrenia.

The potential role of FABP4 in autism spectrum disorder (ASD) presents compelling evidence for its involvement in critical neurobiological processes. FABP4 may significantly influence the production of inflammatory mediators and reactive oxygen species (ROS), both of which are essential factors in neurodegenerative pathways [14]. Moreover, FABP4 is vital for regulating the availability of fatty acids necessary for optimal synaptic function, maintaining membrane fluidity, and ensuring neural integrity [6].

Further reinforcing these findings, studies conducted on animal models have demonstrated that FABP4 knockout (KO) mice exhibit marked deficits in social behavior and an increased density of immature spines, mirroring the phenotype observed in Fragile X Messenger Ribonucleoprotein 1(Fmr1 KO) mice that serve as models for ASD [15]. Additionally, precise analysis of the protein-protein interaction network has uncovered that FABP4 interacts with 102 ASD risk genes, which engage with other crucial proteins. This obscure net of interactions strongly suggests that FABP4-related networks are pivotal in the development and expression of ASD [16].

Notably, in the context of children with intellectual disabilities (ID) as a distinct group of neurodevelopmental disorders, those children with ID showed also significantly reduced levels of FABP4 when compared to their peers who are typically developing. This notable difference presents a compelling opportunity for accurately distinguishing between children with ID and those who are healthy, suggesting that FABP4 may not only contribute to the underlying mechanisms of autism spectrum disorder (ASD) but also play a role in understanding cognitive impairments in children. The innovative work of [17] underscores the pivotal importance of FABP4 in brain cognition. Their findings demonstrate that FABP4 knockout confers strong protection against the production of reactive oxygen species (ROS) and alleviates endoplasmic reticulum (ER) stress. Moreover, the research by [18] provides convincing evidence that adipocyte knockout (AKO) mice are spared from high-fat diet-induced hippocampal pro-inflammatory cytokine expression and corresponding memory deficits. This remarkable protection is linked to a significant reduction in pro-inflammatory signalling, ER stress, and apoptosis while simultaneously promoting neurogenesis, synaptic plasticity, long-term potentiation, and enhanced spatial working memory. Furthermore, A study by [19] puts light on the elaborate molecular mechanisms involved, revealing changes in transcripts related to the regulators of ER to Golgi transport. This adds another layer of understanding to the neuroprotective effects conferred by FABP4 knockout, presenting a compelling case for its critical role in cognitive health.

The findings of this study convincingly demonstrate that FABP4 levels are highly effective in distinguishing between children with (ASD) and those in the control group, as well as between children with (ID) and the control group. However, when we investigated deeper into the potential of FABP4 to differentiate ASD from ID by comparing levels in children with ID to those in children with ASD, the results revealed that FABP4 was significantly less effective in making this distinction. This outcome may be attributed to the shared genetic and behavioral characteristics between (ID) and (ASD) [20]. Additionally, both ASD and ID exhibit overlapping biological mechanisms, including disruptions in synaptic function, oxidative stress, and neuroinflammation [21, 22]. These shared features highlight the complexity of differentiating between these two conditions, underscoring the need for further research in this area.

A growing body of research interestingly demonstrates that childhood neurodevelopmental disorders including Intellectual Disability (ID), (ASD), and Attention-Deficit/Hyperactivity Disorder (ADHD) share specific genetic risk alleles with psychiatric disorders, particularly schizophrenia [23]. Significantly, studies have shown that copy number variants (CNVs) associated with ID are notably enriched in individuals diagnosed with schizophrenia, suggesting that many additional ID-related variants may also enhance the risk for schizophrenia, even though with reduced penetrance [24]. This evidence has led researchers to encourage for a neurodevelopmental continuum model. This model speculates that neurodevelopmental disorders, including schizophrenia, represent a diverse array of outcomes stemming from disrupted or atypical brain development [25].

With respect to the lowered serum levels of FABP4 in ASD, and ID in comparison to their healthy peers, other factors might affect the peripheral level of FABP4 as age and BMI. Although A study by [26] reported a positive association between FABP4 levels and age of participants, particularly in older individuals, we did not observe a significant correlation between FABP4 levels and the age within the studied group. Similarly, several studies found FABP4 circulating levels significantly correlate with the percentage of body fat represented by BMI [27]. However, we found no significant correlation between the levels of FABP4 and BMI, which may implicate that FABP4 may play a role in the brain development independently of body fat and age effect.

A positive family history of neurodevelopmental disorders is a well-established risk factor for (ASD) and Intellectual Disability (ID), indicating an interplay of shared genetic and environmental influences among family members [28, 29]. This heightened vulnerability is complexly linked to shared genetic mutations, including de novo mutations and copy number variations, which play a significant role in the increased prevalence of ASD and ID within families [30, 31]. However, it is noteworthy that our analysis did not reveal a significant difference in FABP4 levels between children with positive versus negative family histories of neurodevelopmental disorders. This may indicate the need for more investigation in more extensive and long-term research.

Emerging evidence highlights the critical role of FABP4 at the fetoplacental interface, significantly impacting pregnancy complications, maternal-fetal immune tolerance, and lipid transportation [32–33]. Notably, several studies have documented elevated levels of FABP4 in preterm infants, particularly those born before 30 weeks of gestation [34–37]. However, our study found no significant difference in FABP4 levels between children with a history of prematurity or NICU admission and those born full-term without postnatal complications across all groups. This finding may stem from the limited number of preterm children in our cohort. Moreover, it raises an interesting possibility that FABP4 might play a key role in brain development regardless of prematurity history. These insights highlight the need for further investigation to explore whether FABP4 could emerge as a reliable predictive biomarker for identifying pregnancies at risk of preterm birth and adverse neurodevelopmental outcomes. Such research could have profound implications for maternal and infant health.

### Limitations

This study suggests that FABP4 may be a biomarker related to the adipobrain axis in autism spectrum disorder (ASD) and intellectual disability (ID). However, it has several limitations. The cross-sectional design prevents causal conclusions, and the focus on a single ethnic group from one centre limits the findings’ broader applicability. The small sample size and lack of overlap between the ASD and control groups may have inflated the high diagnostic performance reported in the receiver operating characteristic (ROC) analysis. The area under the curve (AUC) of 1.00, indicating 100% sensitivity and specificity, should be viewed cautiously due to these cohort-specific factors. Because of the variability in ASD, achieving perfect classification is unlikely without validation in larger, more diverse groups.

While participants fasted for eight hours and had comparable BMI, the study did not assess dietary habits or gastrointestinal issues often seen in children with autism spectrum disorder (ASD), which could influence FABP4 levels.

Most participants in our ASD group showed reduced cognitive performance, leading to an underrepresentation of high-functioning ASD individuals. To improve future research, it is crucial to focus on larger cohorts that differentiate ASD patients by cognitive functioning. This will help clarify the relationship between FABP4 and ASD, as well as other neurodevelopmental impairments.

## Conclusions

Collectively, the findings of this study suggest that FABP4 has the potential to serve as a biomarker for distinguishing children with neurodevelopmental disorders from healthy controls. Lower levels of FABP4 were strongly associated with both ASD and ID, with high sensitivity and specificity in differentiating affected children from controls. Interestingly, the limited ability of FABP4 to discriminate between ASD and ID may support the concept of a neurodevelopmental continuum, reflecting overlapping biological mechanisms and shared pathophysiological features between these conditions rather than entirely distinct disease entities. These findings highlight the potential role of FABP4 in the pathophysiology of neurodevelopmental disorders and underscore the need for further large-scale studies to clarify its diagnostic and therapeutic implications.

### Recommendations and implications

Future research should aim to validate these findings in larger and more diverse populations. It is also important to investigate the specific mechanisms by which FABP4 affects pregnancy outcomes and fetal brain development. Additionally, exploring FABP4 as a potential therapeutic target could lead to new strategies for alleviating pregnancy complications and lowering the risk of neurodevelopmental disorders, including (ASD).

## Data Availability

All data generated or analyzed during this study are included in this published article or are available from the corresponding author upon reasonable request.

## Abbreviations

ADHD: Attention deficit hyperactivity disorder
AKO: Adipocyte knockout
ANOVA: Analysis of Variance
aP2: Adipocyte protein 2
ASD: Autism spectrum disorder
AUC: Area under the curve
BMI: Body mass index
CARS: Childhood Autism Rating Scale
CNVs: Copy number variants
DSM- V: Diagnostic and Statistical Manual of Mental Disorders, 5th Edition
ELISA: Enzyme-Linked Immunosorbent Assay
ER: Endoplasmic reticulum
FABP4: Fatty acid-binding protein 4
HRP: Horseradish Peroxidase
ID: Intellectual disability
IQ: Intelligence Quotient
IQR: Interquartile range
KO: Knock out
NDDs: Neurodevelopmental disorders
NICU: Neonatal intensive care unit
OD: Optical Density
ROS: Reactive oxygen species
SD: Standard deviation
SPSS: Statistical Package for Social Sciences
WAT: White adipose tissue
